# Evaluation on immediate analgesic efficacy and safety of Kai-Hou-Jian spray (children’s type) in treating sore throat caused by acute pharyngitis and tonsillitis in children: study protocol for a randomized controlled trial

**DOI:** 10.1186/s13063-021-05148-1

**Published:** 2021-03-18

**Authors:** Yan-ning Ma, Cheng-liang Zhong, Si-yuan Hu, Qiu-han Cai, Sheng-xuan Guo

**Affiliations:** 1 Department of Clinical Trial Center, First Teaching Hospital of Tianjin University of Traditional Chinese Medicine, National Clinical Research Center for Chinese Medicine Acupuncture and Moxibustion, No. 88 Changling Street, Xiqing District, Tianjin, 300193 China; 2 Department of Pediatrics, First Teaching Hospital of Tianjin University of Traditional Chinese Medicine, National Clinical Research Center for Chinese Medicine Acupuncture and Moxibustion, No. 88 Changling Street, Xiqing District, Tianjin, 300193 China

**Keywords:** Acute pharyngitis/tonsillitis, Kai-Hou-Jian spray, Randomized controlled trial, Sore throat, Traditional Chinese medicine

## Abstract

**Background:**

Acute pharyngitis and tonsillitis are common respiratory diseases for which children seek medical care. Their main clinical manifestation is sore throat which interferes with patients’ quality of life. However, there is no proven effective or safe method to treat it. It is necessary to find an excellent strategy to reduce sore throat and reduce the burden of acute illness. We designed the randomized controlled trial with the characteristics of traditional Chinese medicine (TCM) to determine the clinical positioning of Kai-Hou-Jian spray (children’s type) (KHJS) through evidence-based research. This trial aims to evaluate the immediate analgesic efficacy of KHJS on sore throat caused by acute pharyngitis and tonsillitis (wind-heat syndrome/heat exuberance in lung and stomach syndrome) in children and to observe its safety.

**Methods/design:**

This is a prospective, multicenter, randomized, double-blind, parallel-group, placebo-controlled trial. It will include 240 children with acute pharyngitis/tonsillitis from 7 study sites across China. All participants are randomly assigned to two parallel treatment groups, one with KHJS and the other with placebo sprays, for 5 consecutive days. The primary outcome is the time of analgesic onset. Secondary outcomes include duration of analgesic effect, area under time curve of 0–3 h Wong-Baker FACES Pain Rating Scale (WBS) score (AUC0-3 h), rate of analgesic onset, rate of disappearance of sore throat, changes of WBS score (in days), effective rate of pharyngeal signs, and effective rate of TCM syndrome. The incidence of adverse events during the trial is the primary safety outcome. In addition, vital signs and laboratory tests before and after medication are monitored.

**Discussion:**

To our knowledge, this will be the first clinical trial to explore the immediate analgesic efficacy of a Chinese patent medicine spray for acute pharyngitis/tonsillitis induced sore throat in children in a multicenter, randomized, double-blinded, parallel-group, placebo-controlled manner. Not only might it prove the efficacy and safety of KHJS in the treatment of sore throat caused by acute pharyngitis/tonsillitis in children, but it might also provide evidence for the treatment of acute sore throat with Chinese herbal medicine.

**Trial registration:**

A multicenter, randomized, double-blind, very low-dose, parallel controlled trial for the immediate analgesic effect and safety of Kai-Hou- Jian spray (children's type) in the treatment of sore throat caused by acute pharyngitis and tonsillitis in children. Chinese Clinical Trial Registry ChiCTR2000031599. Registered on 5 April 2020

**Supplementary Information:**

The online version contains supplementary material available at 10.1186/s13063-021-05148-1.

## Background

Acute pharyngitis and tonsillitis are common respiratory diseases for which children seek medical care and they are sometimes referred to together as acute sore throat [1].Seventy to 95% of children with acute sore throat are caused by virus, mainly respiratory virus, while 15–30% are caused by bacterial, such as β hemolytic group A *Streptococcus* [2–4]. Systemic analgesia or soothing measures such as gargling with warm salt water are often used to treat sore throat caused by acute pharyngitis/tonsillitis, but the relief is short and some systemic analgesic agents have been shown to have some adverse effects. For children with bacterial pharyngitis/tonsillitis, antibiotic therapy should be added to prevent complications and the spread of infection, but antibiotics are less effective in reducing pain [5, 6]. Sore throat seriously affects the life quality of children and their families, so it is necessary to find an excellent strategy to reduce symptoms and reduce the burden of acute illness.

Kai-Hou-Jian spray (children’s type) (KHJS), which has been on the market for more than 10 years, is a Chinese patent medicine produced by Guizhou Sanli Pharmaceutical Co., Ltd. (Guizhou, China). It has been authorized by the China State Food and Drug Administration (Drug Approval number 20025142) and is commonly used in the clinical treatment of pharyngitis, tonsillitis, sore throat, stomatitis, and so on. The prescription of KHJS comes from Miao medicine experience in Guizhou. It is mainly composed of *Ba Zhao Jin Long [Ardisia crispa (Thunb.) A.DC.]*, *Shan Dou Gen [sophorae tonkinensis radix etrhizoma]*, *Chan Tui [cicadaeperiostracum]*, *Bo He Nao [l-menthol]*. In the theory of traditional Chinese medicine (TCM), KHJS have the functions of clearing heat and removing toxicity and relieving swelling and pain. The pharmacodynamic test showed that KHJS could inhibit the auricular inflammation of mice caused by xylene, relieve the fever of rats caused by typhoid and paratyphoid triple vaccine, and reduce the number of writhing of mice caused by acetic acid. In vitro antiviral and bacteriostatic tests showed that KHJS has a wide range of inhibitory effects on influenza A virus, Gram-positive bacteria, and Gram-negative bacteria [7]. According to the data, the incidence of adverse reactions of KHJS from 2017 to 2019 is < 0.01%, which belongs to the “very rare” category [8].

There have been previous studies in humans on the use of KHJS in the treatment of acute pharyngitis /tonsillitis, but there are some problems, such as too small sample size and not using double-blind methods. Moreover, these studies take the overall improvement of several TCM symptoms as the primary outcome, which is lack of objectivity and science to a certain extent [9, 10]. We designed this prospective, multicenter, randomized, double-blind, parallel-group, placebo-controlled trial to seek a proven effective and safe way to reduce the pain caused by acute pharyngitis /tonsillitis. The objective of this trial is to evaluate the immediate analgesic efficacy of KHJS on sore throat caused by acute pharyngitis /tonsillitis (wind-heat syndrome/heat exuberance in lung and stomach syndrome) in children, and to observe its clinical safety.

## Methods/design

**Fig. 1:**
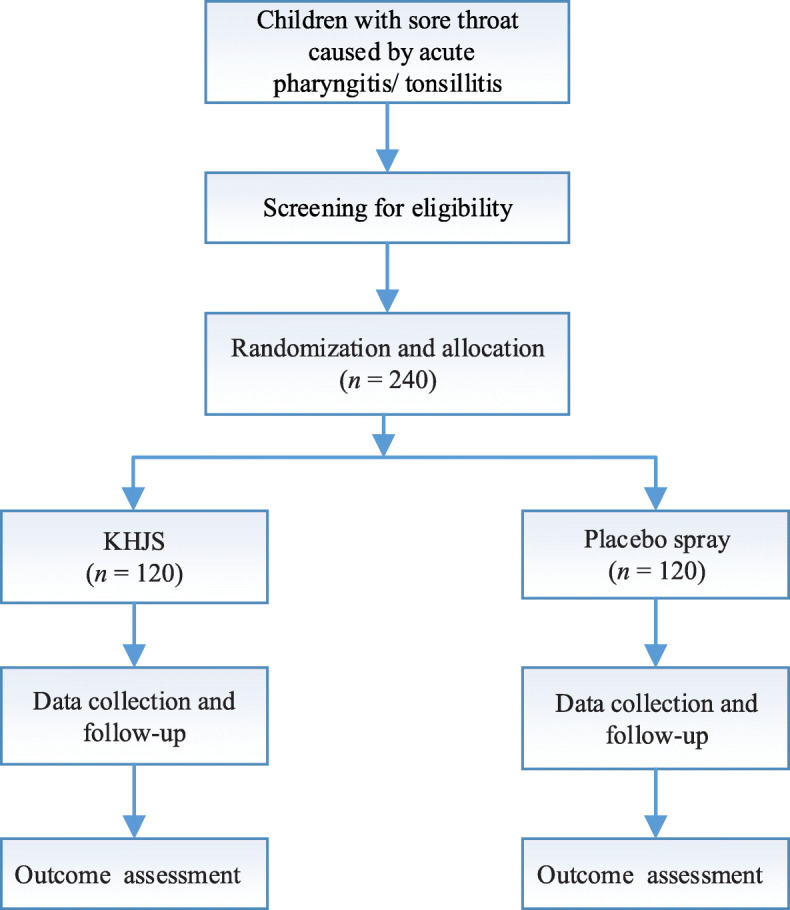
Study flow chart

This is a prospective, multicenter, randomized, double-blinded, parallel-group, placebo-controlled trial of KHJS versus placebo spray for acute pharyngitis/tonsillitis in children. Supported by ' Evidence-based study on the clinical location of KHJS '(NO.2018YFC1708106), Major Project ' Modernization of TCM ' of Ministry of Science and Technology of the People's Republic of China. In addition, it has been registered on the Chinese Clinical Trial Registry (ChiCTR2000031599). The study protocol is conducted in accordance with the Declaration of Helsinki, the code of Good Clinical Practice, and the guidelines of the International Conference on Harmonisation. Recruitment is scheduled to occur from June 2020 until December 2020. Figure 1 shows the flow chart of the trial.

### Patient population and setting

The diagnosis of acute pharyngitis/tonsillitis is made by a qualified pediatrician with reference to *Practical pediatric otolaryngology*, *Clinical pediatrics*, and *ZhuFutang practical pediatrics* [11–13]. It is mainly summarized as the onset of urgent, first dry pharynx, and then pharynx pain, pharynx or tonsil congestion, swelling or even suppuration, or accompanied by cervical lymph node enlargement, tenderness, as well as also accompanied by fever, headache, poor appetite, and other systemic symptoms. Syndrome differentiation standard of wind-heat syndrome/heat exuberance in lung and stomach syndrome of pharyngitis/tonsillitis is formulated according to the *Guidelines for the diagnosis and treatment of common diseases in traditional Chinese otorhinolaryngology* [14] and *Guidelines for the diagnosis and treatment of common diseases in pediatrics of traditional Chinese medicine* [15].

A total of 240 children of either sex, aged 4–12 years, will be enrolled from 7 study sites across China: (1) the First Teaching Hospital of Tianjin University of TCM, (2) Dongzhimen Hospital, Beijing University of Chinese Medicine, (3) Xiangya Hospital, Central South University, (4) Nanjing Children’s Hospital, (5) Tianjin Medical University General Hospital, (6) the First Affiliated Hospital of Hunan University of Traditional Chinese Medicine, and (7) Children’s Hospital of Chongqing Medical University.

#### Inclusion criteria

1. Clinical diagnosis of acute pharyngitis/acute tonsillitis;
2. Wind-heat syndrome or heat exuberance in lung and stomach syndrome in TCM;
3. Wong-Baker FACES Pain Rating Scale (WBS) score of sore throat with swallowing ≥ 4, body temperature < 38.5°C;
4. Aged 4 to 12 years, can use WBS correctly;
5. All patients (≥ 8 years old) and their guardians should sign the informed consent before entering the study, and children under 8 years old should sign the informed consent by their guardians.

#### Exclusion criteria

1. At the first diagnosis, the child’s condition is more serious, and he/she suffers from poor mental state, irritability, headache, or limb muscle pain, and needs antipyretic analgesics;
2. Children or their parents/caregivers have difficulty understanding or cooperating with the use of WBS;
3. Children with peritonsillar or retropharyngeal abscesses, infectious mononucleosis, measles, or scarlet fever;
4. Children with severe primary diseases of heart, lung, liver, kidney, metabolism, hematopoietic, immune, nervous, and spiritual systems;
5. Children with acute laryngitis, otitis media, bronchitis, pneumonia, and other complications;
6. Allergic to components of KHJS, or antipyretic analgesics;
7. Those who had taken acetaminophen, ibuprofen, or other antipyretic analgesic within 6 h before enrollment;
8. Unfixed residence, children’s apparent inability to cooperate with the study, and other conditions deemed inappropriate by the researchers.

#### Withdrawal criteria

1. Children with severe complications;
2. Children with no improvement or aggravation after 3 days of treatment;
3. Children with anaphylaxis or serious adverse events who are judged by the physician to be discontinued;
4. Children with poor medication compliance;
5. Children who break blind procedure for a variety of reasons;
6. Children participating in the randomization who have seriously violated the inclusion or exclusion criteria.
7. Children who are unwilling or unable to continue the trial for a variety of reasons and request to withdraw from the trial.
8. Lost to follow-up children who are not explicitly asked to withdraw from the trial but are no longer receiving experimental drug or testing.

#### Termination criteria

1. When a serious safety event occurs in the clinical trial.
2. When it is found that there is a major error in the clinical trial protocol, or there is a serious deviation in the implementation of the protocol.
3. In the course of the experiment, it is found that the experimental drug has poor therapeutic effect and no clinical application value.
4. The ethics committee called for the trial to be suspended or terminated.
5. The competent administrative department canceled this trial.

### Randomization, concealment, and masking

Randomization (1:1) will be stratified by study site, and the randomization list is generated by an independent statistician using SAS v 9.2. Two hundred forty children are divided into 40 blocks with the block size as 6. The documents of random process and randomization list will be enclosed in an opaque envelop. Participants, researchers, and coordinators together with statisticians were not aware of the allocation of the patient and remained unaware until trial completion. All study sites will also receive opaque envelopes containing the experimental drug’s website, login ID, password, instructions, security information, and treatment recommendations, which the researchers should open in an emergency.

In this trial, we use a very low dose (5%) of KHJS solution as a placebo spray. It contains 0.525 g/15 ml of crude herbs, while KHJS contains 10.05 g/15 ml crude herbs. Both the KHJSs and placebo sprays are manufactured by Guizhou Sanli Pharmaceutical Co., Ltd. with the same specification, appearance and flavor. These sprays are coded, packed, and labeled based on randomization list. Each center has an independent drug administrator responsible for the storage, distribution, recovery, and documentation of all experimental drugs.

### Interventions

All participants are randomly allocated to one of the two parallel treatment groups. Spray the pharyngeal region with KHJS or placebo spray in a dose of 5 sprays for ages 4 to 6 and 7 sprays for ages 7 to 12. Depending on the condition of the sore throat, the spray can be reused every 3 to 6 h, but not more than 8 times every 24 h. Treatment is planned for 5 consecutive days, but patients whose sore throat disappears may terminate treatment early.

### Concomitant treatments and forbidden medication

Children with indications of bacterial infection should be treated with antibiotics [16]. Antiviral chemicals are allowed if a child is infected with virus. Children with fever can adopt physical cooling measures such as cooling paste and warm water bath. Antipyretic analgesic should not be used until 3 h after the first dose. Other pharyngeal local medications or system medications with antipyretic detoxification effect are prohibited. All concomitant treatments or medication administered must be recorded carefully in case report form (CRF) and subject medical record, including the name, duration of administration, dosage, and cause of administration. It is also necessary to determine whether concomitant medication affects the efficacy of experimental drug.

### Outcome measures

#### Primary outcome

The primary outcome is the time of analgesic onset. Analgesic onset is based on WBS score of sore throat (swallowing pain, same below) intensity, and defined as the reduction of WBS score ≥ 1 grade after the first dose. The WBS uses 6 different faces from smile to crying to describe the pain intensity of children aged 3 and above, from no hurt (0 point) to hurts worst (10 point), with a 2-point interval. It does not need specific cultural background, is easy to grasp, and has a good correlation with other commonly used scales [17, 18]. Children will be asked to swallow and to choose an expression to show how much their throat hurts now. In order to obtain the data of primary outcome, the children will be evaluated 12 times for WBS score (baseline and 5, 10, 20, 30, 40, 50, 60, 90, 120, 150, and 180 min after the first dose) by researchers in the hospital.

#### Secondary outcome

We will also collect data for the following additional outcomes: duration of analgesic effect, area under time curve of 0–3 h WBS score (AUC0-3 h), rate of analgesic onset, rate of disappearance of sore throat, changes of WBS score (in days), effective rate of pharyngeal signs, and effective rate of TCM syndrome.

The duration of analgesic effect, AUC0-3 h, and the rate of analgesic onset are all obtained from the WBS scores in first 3 h. The rate of analgesic onset will be evaluated at 30 and 60 min after the first dose. Rate of disappearance of sore throat will be evaluated at full 3 and 5 days. Disappearance of sore throat is defined as a reduction in WBS score to 0. Changes of WBS score will be evaluated at full 1, 2, 3, 4, and 5 days conducted at home by the child’s parents/caregiver.

The data of effective rate of pharyngeal signs and effective rate of TCM syndrome are collected by the symptoms and signs scale based on TCM syndromes (Table 1) compiled according to some guidelines [14, 15, 19, 20]. Both of the effective rate of pharyngeal signs and effective rate of TCM syndrome will be evaluated at baseline and full 5 days. Effectiveness of pharyngeal signs means the reduction of total score range from baseline to finish ≥ 50%. The effectiveness of TCM syndrome is defined as the decline rate of TCM syndrome score ≥ 50% after medication.

**Table 1:** Symptoms and signs scale based on TCM syndromes

**TCM main syndrome**		**Score grading**					
		**0**	**1**	**2**	**3**	**4**	**5**
Sore throat		WBS 0	WBS 2	WBS 4	WBS 6	WBS 8	WBS 10
**TCM minor syndrome**		**Score grading**					
		**0**	**1**	**2**	**3**		
**Wind-heat syndrome**	Fever	No	37.3–38 °C	38.1–38.9 °C	≥ 39 °C		
	Headache	No	Yes				
	Afraid of the wind	No	Yes				
	dryness and heat of pharyngeal	No	Yes				
	Cough or with yellow sputum	No	Yes				
**Heat exuberance in lung and stomach syndrome**	Fever	No	37.3–38 °C	38.1–38.9 °C	≥ 39 °C		
	Cough or with yellow sputum	No	Yes				
	Thirst	No	Yes				
	Bad breath	No	Yes				
	Abdominal distension	No	Yes				
	Dry stool	No	Yes				
	Yellow-colored urine	No	Yes				
**Pharyngeal signs**		**Score grading**					
		**0**	**1**				
**Wind-heat syndrome**	The pharyngeal mucosa is bright red and swollen	No	Yes				
	Tonsil is red and swollen, without pus	No	Yes				
	The lymph nodes are swollen and painful under the jaw	No	Yes				
**Heat exuberance in lung and stomach syndrome**	The pharyngeal mucosa is red and swollen	No	Yes				
	Tonsil is red and swollen, or companion pus point	No	Yes				
	The lymph nodes are swollen and painful under the jaw	No	Yes				

### Safety outcome measures

The incidence of adverse events (AEs) during the trial is the primary safety outcome measure. In addition, vital signs and laboratory tests before and after medication are monitored. Vital signs refer to temperature, heart rate, respiration, and blood pressure. Laboratory tests include blood, urine, hepatic and renal functions, electrocardiography, and C-reactive protein (inspect only at pre-medication). If the child is unable to cooperate or strongly disagree with the test for non-clinical need, such as hepatic and renal functions, the researcher must respect the patient’s wishes and record the reason for missing safety data on subject medical record with parents’ signature. All abnormal safety measure should be followed up until recover or return to pre-medication levels.

#### Record, assessment, management, and report of AE

The researcher should truthfully record all AEs related to children in subject medical record and CRF, including occurrence time, end time, duration, severity, therapeutic measures, outcome, and relationship with experimental drug.

The severity of AE can be graded according to the National Cancer Institute Common Terminology Criteria for Adverse Events (NGI-GTCAE) V.5.0 [21]. If it does not apply to certain specific AEs, the researchers will use the 5-level criteria from mild to death. Relationships between AE and experimental drug can be divided into definitely related, probably related, possibly related, probably not related, need further evaluated, and unknown. The first 4 cases will be considered as adverse drug reactions according to *adverse drug reactions report and monitoring manual* [22].

When AEs occur, researchers should make timely response measures, if necessary, can break blind procedure, and report to the sponsor and the clinical research associate. The sponsor is responsible for monitoring and reporting safety information and shall promptly report serious adverse events to all researchers, clinical trial institutions, ethics committees, and adverse reaction monitoring agency. Report of AEs shall be in accordance with the *Rapid reporting standards and procedures for safety data during drug clinical trials* issued by China’s center for drug evaluation, National Medical Products Administration (NMPA) [23].

### Schedule of study procedures

**Fig. 2:**
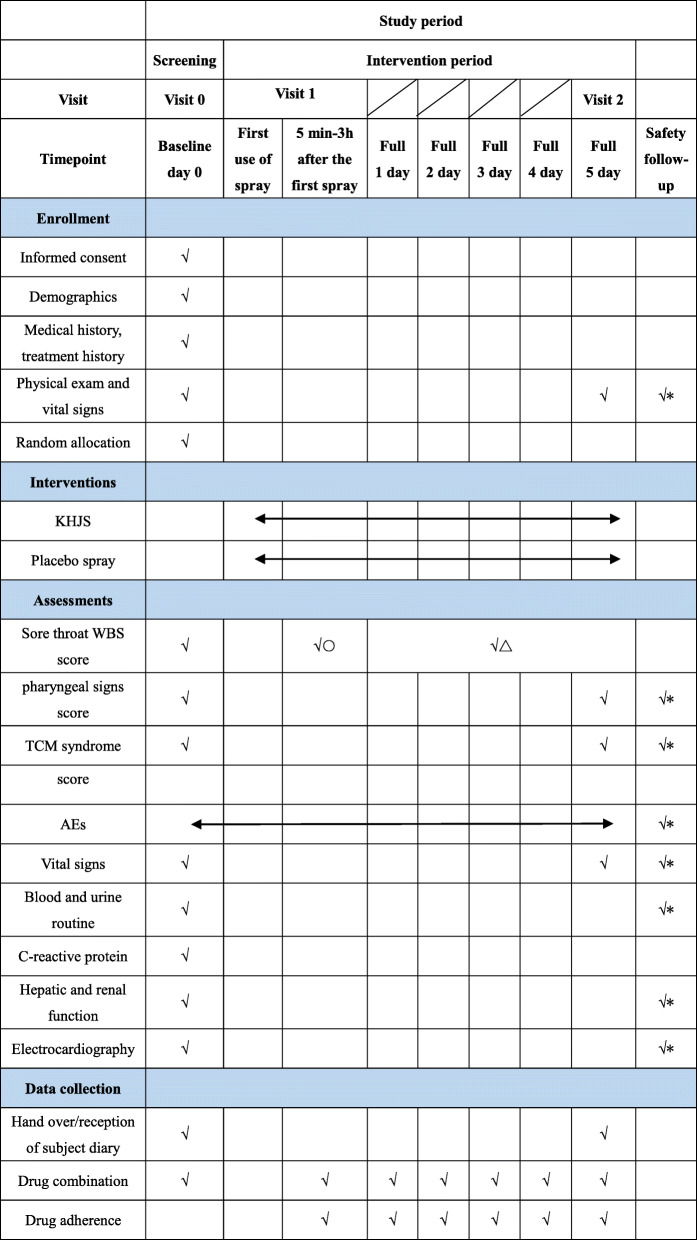
Schedule of study procedures. √○, children will be evaluated for WBS score (5, 10, 20, 30, 40, 50, 60, 90, 120, 150, and 180 min after the first dose) by researchers in the hospital; √△, children will be evaluated for WBS score (full 1, 2, 3, 4, 5 days) by the child’s parents/caregiver at home; √*, if there is an AE, the patient needs to be followed up for safety information till reach the endpoint of event

Figure 2 is the schedule of screening, enrollment, intervention, assessments, and data collection.

### Sample size calculation

Based on the primary outcome, the software PASS15.0 is used to calculate the sample size. According to relevant literature and previous studies [24], the median time of producing analgesic effect in the experimental group is 10 min, and that in the control group is 20 min. Set α as 0.025, β as 0.2, and the optimal bound value as 0. Taking into account the 10% loss to follow-up rate, the final estimated sample size is 105 cases per group. After comprehensive consideration, the final decision of each group of 120 cases, a total of 240 cases.

### Data collection, management, monitoring, and auditing

Before the start of the trial, each study site should hold the project kick-off meeting respectively to strengthen the researchers and quality control personnel to master the trial plan and the operation technology of the key nodes. In addition, researchers should be trained specifically in the methodology of indicator evaluation.

In this study, we designed subject medical record and subject diary to record the first-hand clinical trial data of children. Researchers or clinical research coordinator (CRC) should collect relevant information and record it on the subject medical record timely, completely, and accurately. The subject diary is used to record the WBS score and the experimental drug use of the children. In order to improve compliance of the children until completion of the trial, we will strengthen patient management by assigning investigators one-on-one follow-up.

The data manager will design electronic CRF according to the trial plan and provide an input guidance. The trained CRC shall enter information from the subject medical record and subject diary into the electronic CRF within 3 days of each visit. This trial is designed to encode AEs and concomitant medication on electronic CRF. Medical coders coded the AEs according to MedDRA 22.0, and the concomitant medication code was classified by WHO ATC (World Health Organization, Anatomical Therapeutic Chemical classification). The database setup follows CDISC standard, linked with electronic CRF and relying on electronic data capture system which has logic verification and data capture function. Data manager will clean and verify the database according to the data verification plan previously written after finishing data collection and suspicious data to researchers circularly till all questions are answered. After that, researchers, data managers, and statisticians will hold a data verification meeting under the blind state for discussing data process and the partition of data sets. Once the agreement is reached, the database is locked and no modifications are allowed thereafter. After the completion of the trial, the documents involved in the whole trial shall be kept until at least 2 years after the termination of the clinical trial.

Since KHJS has more than 10 years of good clinical experience, and considering the funding and time constraints of the project, there will be no interim analysis. To ensure the quality of the clinical trial, an independent expert committee consisting of clinical experts, statisticians, pharmaceutical experts, pharmacology, and toxicology experts will be established to monitor and evaluate patient safety and efficacy data in a blind manner. It will also assess whether participants received good clinical care and safety issues explained and properly resolved, as well as recommendations for protocol modifications or even recommendations and decisions to terminate the study based on efficacy and safety.

Auditors independent of the researchers and sponsors will conduct monthly reviews to systematically review the activities and documents related to the study to evaluate whether the study was conducted in accordance with the study protocol, GCP, and relevant regulatory requirements and whether the trial data is recorded in a timely, true, accurate, and complete manner.

### Statistical analysis

In this experiment, the superiority trial design is adopted. Full analysis set (FAS) included all children who were randomized, received at least one spray of medication, and had at least one visit record. Per-protocol set (PPS) includes only patients who fulfill the protocol. All subjects who have taken the experimental drug will have their data covered by the safety set (SS) if the safety results are recorded. FAS and PPS are selected for effectiveness evaluation, and SS is selected for safety evaluation. If there are missing values in the primary outcome, the method of carrying forward the last observation data to the final results of the trial is adopted. An independent statistician will use SAS v 9.2 for statistical analysis. All hypothesis tests are conducted by two-sided test, and the overall comparison inspection level is 0.05 of α.

Survival data will be statistically described by median, upper quartile, lower quartile survival time, and 95% confidence interval (95% CI), and survival curve will be made. The comparison between groups is checked by log-rank test. If the influence of baseline factors is considered, Cox regression analysis is adopted. For quantitative data, the number of cases, mean, standard deviation, minimum, median, maximum, upper quartile, lower quartile, and 95% CI are used to describe the data. *T* test or paired *t* test is used for statistical analysis of data before and after treatment in two groups or within groups. If the influence of center or other confounding factors are considered, analysis of covariance is used.

For qualitative data, frequency table, percentage, or composition ratio is used to describe the data. For statistical analysis of data before and after treatment in two groups or within groups, *χ*^2^ test, Fisher’s exact probability method, Wilcoxon rank sum test, or Wilcoxon’s sign rank sum test is used. For the comparison of two categories and ranked data, if the influence of center or other factors are considered, CMHχ^2^ test is adopted. If the influence of confounding factors is considered, logistic regression analysis is adopted.

### Ethical requirement

This study protocol has been submitted to the ethics committee of First Teaching Hospital of Tianjin University of TCM, the unit of clinical trial leader to get approval. It will also be submitted to other centers to get consensus. If there is any significant modification of the study protocol, the revised protocol, and the informed consent, CRF, researcher manual and other relevant documents involved in the modification should be resubmitted to the ethics committee for approval. The researcher conducting the study should be a senior attending physician or above and have received training in the relevant experimental techniques. In the event of injury or death related to the trial, the sponsor will be responsible for the treatment costs and financial compensation. Children who have not been cured after the end of the trial may continue to be treated by other medical methods at the expense of themselves. If the adverse reaction caused by the experimental drug occurs in patients during the trial, and the adverse reaction remains uncured after the end of the administration cycle, the sponsor shall bear the cost of treatment. Only the researchers and monitors involved in this trial have access to subject medical records, and the subjects’ data will not be used in other ancillary studies. The ethics committee and the pharmaceuticals supervisory and administrative departments shall have the right to consult the relevant research records of this project. In order to protect the privacy of the children during data processing, their identity information will be omitted. Both during and after the study, the subject medical records will be kept under strict security. Before screening, the researcher should explain the details of the project to the guardian and the child in plain language, so that they can fully understand and have enough time to consider. According to Article 20 of the *General Rules of the Civil Law of the People’s Republic of China*, “A minor who has not reached the age of eight yet is a person having no capacity for civil conduct and shall be represented in the performance of civil juristic acts by his or her agent ad litem”. All patients (≥ 8 years old) and their guardians should sign the informed consent before entering the study, and children under 8 years old should sign the informed consent by their guardians. Researchers should make children aware that participation is entirely voluntary and that they have the right to withdraw from the study at any time. The study protocol is based on the SPIRIT checklist [25]. The results of this study will be submitted to peer-reviewed journals.

## Discussion

TCM has a long history and has advantages in the treatment of many diseases, but it is not recommended by the international medical community due to the lack of evidence in related fields. After literature search and analysis, we found that the previous randomized controlled trials of Chinese patent medicine sprays used in children with acute pharyngitis/tonsillitis had poor methodological quality. KHJS has been on the market in China for more than a decade and has been widely used in the treatment of clinical throat diseases. We designed the prospective, multicenter, randomized, double-blind, parallel-group, placebo-controlled trial with the characteristics of TCM to determine the clinical positioning of KHJS through evidence-based research. After the high-level design meeting and feasibility study meeting held by multidisciplinary experts including pediatricians, statisticians, and methodology experts, the trial plan was finally formulated.

To our knowledge, this will be the first clinical trial to explore the immediate analgesic efficacy of a Chinese patent medicine spray for acute pharyngitis/tonsillitis induced sore throat in children in a multicenter, randomized, double-blinded, parallel-group, placebo-controlled manner. This study can not only prove the efficacy and safety of KHJS in the treatment of sore throat caused by acute pharyngitis/tonsillitis in children, but also provide evidence for the treatment of acute sore throat with Chinese herbal medicine.

Considering that throat sample culture is not routinely used in outpatient practice and may increase barriers to study entry, we did not take this method in this trial plan. Compared with using positive drug as control, choosing placebo as control can better test the absolute efficacy and safety of experimental drug in a randomized controlled trial. However, in clinical trials of Chinese patent medicines, especially liquid compounds, it is difficult to develop placebos that match the color, smell and taste of the experimental drugs. In order to better implement the blind method, we refer to relevant research data [26] and finally use 5% of the total dose of KHJS solution as placebo, but this method may interfere with the safety evaluation to some extent.

## Trial status

Protocol version 1.0, 15 January 2020. Recruitment is scheduled to occur from June 2020 until December 2020.

## Supplementary Information

**Additional file 1.** SPIRIT 2013 Checklist: Recommended items to address in a clinical trial protocol and related documents*.

## Data Availability

The results will be published in a peer-reviewed journal and poster or presented orally at conferences. Data sets generated or analyzed in this study will be provided by the corresponding authors upon reasonable request.

## Abbreviations

KHJS: Kai-Hou-Jian spray (children’s type)
TCM: Traditional Chinese medicine
WBS: Wong-Baker FACES Pain Rating Scale
CRF: Case report form
AEs: Adverse events
NMPA: National Medical Products Administration
CRC: Researchers or clinical research coordinator
FAS: Full analysis set
PPS: Per-protocol set
SS: Safety set
95%CI: 95% confidence interval
